# A negative biological Indian Ocean dipole event in 2022

**DOI:** 10.1038/s41598-024-51347-6

**Published:** 2024-01-11

**Authors:** Wei Shi, Menghua Wang

**Affiliations:** 1 NOAA National Environmental Satellite, Data, and Information Service, Center for Satellite Applications and Research, E/RA3, 5830 University Research Ct., College Park, MD 20740 USA; 2 CIRA at Colorado State University, Fort Collins, CO 80523 USA

**Keywords:** Ocean sciences, Marine biology, Physical oceanography

## Abstract

The biological dipole mode index (BDMI) showed a negative biological Indian Ocean dipole (BIOD) event occurred in the Equatorial Indian Ocean with the corresponding BIOD index BDMI^(Ratio)^ at − 0.31 in October 2022. The chlorophyll-a (Chl-a) ratio (or Chl-a anomaly) between Chl-a in October 2022 and October Chl-a climatology from the Visible Infrared Imaging Radiometer Suite (VIIRS) showed negative dipolar features with the depressed and enhanced Chl-a in the east and west IOD zones, respectively. During this negative BIOD event, Chl-a ratio dropped to ~ 0.4–0.5 in the offshore region of the west Sumatra Coast in the east IOD zone, while it increased to ~ 1.5–1.6 in the northern west IOD zone. Temporal variations of the longitudinal averaged Chl-a ratio and the 20 °C isothermal (ISO20) depth anomaly generally coincided and collocated with each other. The positive and negative BIOD events in 2019 and 2022, respectively, were attributed to the nutrient dynamics driven by the physical dynamics in these two phases of IOD events. In the negative BIOD event in 2022, the depressed Chl-a in the east IOD zone was attributed to low sea surface nutrient levels due to dampened upwelling and deepened thermocline, while anomalously high Chl-a in the west IOD zone were driven by higher sea surface nutrient concentrations caused by the surface water divergence and shoaling thermocline.

## Introduction

One of the most important phenomena of the Equatorial Indian Ocean (EIO) is the recurrence of the Indian Ocean Dipole (IOD) events^1,2^. Driven by the intra-seasonal oscillation of wind along the west Sumatra Coast^3,4^ due to the anomalous inter-hemisphere pressure gradient (IHPG)^5^, the notable observed feature of the IOD event is the existence of dipolar sea surface temperature (SST) anomaly in the eastern and western EIO regions, i.e., higher-than-normal SST in the eastern EIO and lower-than-normal SST in western EIO or vice versa. The upper ocean variability in the tropical Indian Ocean is strongly modulated by the IOD events. The IOD accounts for about 12% of the SST variability in the Indian Ocean^1^. In fact, the dominant mode of the interannual variability in the subsurface tropical Indian Ocean is governed by the dipole events^6^.

Based on the dipolar SST performance in the EIO, the dipole mode index (DMI) was developed to identify the IOD event, characterize and quantify the development and the strength of the IOD events^1^. Specifically, a positive IOD event represents SST cooling, lower sea level, and shallow thermocline caused by the stronger wind-driven coastal upwelling in the east IOD pole^3,4,7,8^, as well as the warmer-than-normal SST and deepening thermocline in the west IOD pole^8,9^. Studies show that some of the IOD events are related to the variability of the tropical Pacific variability such as the El Niño Southern Oscillation (ENSO)^10–12^. In the last two and half decades, there were a couple of important positive and negative IOD events. Specifically, two severe IOD events occurred with DMI at + 1.279 °C in November 1997 and + 0.964 °C in October 2019 (https://psl.noaa.gov/), while an extreme negative IOD event occurred in 2016^13^.

Broadly, the IOD event is not only a basin-wide physical fluctuation of the atmosphere and ocean in the EIO, but also a biological and biogeochemical variability in the region. Indeed, Shi and Wang^7^ reported a biological IOD (BIOD) event in 2019. The 2019 positive BIOD event led to anomalously enhanced chlorophyll-a (Chl-a) concentration in the east IOD zone and depressed biological activity in the west IOD zone^7,8^. This ocean biological variability is attributed to the nutrient dynamics driven by the enhanced upwelling in the east and the stronger convergence of the surface waters in the west^7^.

Based on the dipolar Chl-a anomalies in the east and west IOD zones during the IOD events, the concept of Biological Dipole Mode Indices (BDMIs) has been proposed to characterize and quantify the development and strength of the BIOD events^8^. In comparison to DMI, the BDMIs reflect the thermocline dynamics due to the intrinsic linkage between the nutrient supply for phytoplankton growth and the thermocline variability. The BDMIs and DMI together provide a better understanding of the ocean processes for both surface and subsurface in the EIO region^7,8^.

Numerous studies have investigated the major positive IOD events in the last two and half decades to address the IOD mechanism and driving forces^2,4,11,14,15^, physical impacts on global ocean and atmosphere^16–19^, as well as the biological, environmental and socioeconomic impacts^7,20–23^.

However, few researches have been conducted to study the negative IOD events, and none has been ever focused on the negative BIOD event. A notable negative IOD/BIOD event occurred in the 2022 autumn that is worth for further investigation. Therefore, we have two objectives of this study. First, following the report of a strong positive BIOD event in 2019^7^, we use this negative BIOD event as a complementary example to show the variability of the entire IOD/BIOD cycle covering both positive and negative phases of the IOD events. Second, we further demonstrate the concept of the BDMI^8^, which can be used to characterize the temporal variability of the biological activities under the negative IOD event, and assess its relationship with the corresponding physical variability, i.e., SST, thermocline dynamics, etc., in the EIO region.

## Data and methods

Similar to the study of the 2019 positive BIOD event^7^, multiple datasets, i.e., satellite derived Chl-a, SST, DMI, subsurface temperature and vertical velocity are used in this study to conduct this investigation. The negative BIOD event is quantified and characterized, and the driving mechanism and the corresponding change in the subsurface are further explored.

In this study, global ocean color product data from the Visible Infrared Imaging Radiometer Suites (VIIRS) onboard the Suomi National Polar-orbiting Partnership (SNPP) are available at the NOAA CoastWatch (https://coastwatch.noaa.gov/). The wind, SST, and vertical velocity data were obtained from NOAA Physical Science Laboratory (https://psl.noaa.gov/).

As a surrogate for the ocean biological activity, the monthly composite Chl-a product^24^ from VIIRS-SNPP^25^ between 2012 and early 2023 are used. The VIIRS ocean color products^26^ have been well calibrated^27,28^ and validated^26,29,30^, as well as being routinely monitored (https://www.star.nesdis.noaa.gov/socd/mecb/color/) using the in situ measurements such as the Marine Optical Buoy (MOBY)^31^, the Aerosol Robotic Network Ocean Color (AERONET-OC)^32^, and annual dedicated VIIRS calibration and validation (Cal/Val) cruises.

To remove the seasonal variability of Chl-a, the monthly climatology Chl-a in the EIO were derived from VIIRS-SNPP observations since February 2012. Shi and Wang^8^ showed that the BDMI^(Ratio)^, which is the ratio between the monthly Chl-a and the corresponding monthly climatology Chl-a, and BDMI^(Diff)^, which is the difference between the monthly Chl-a and the corresponding monthly climatology Chl-a in the two IOD zones, have similar performance in characterizing the BIOD events. Considering that Chl-a values range widely between < 0.1 mg m^−3^ and > 5 mg m^−3^ in the EIO, Chl-a anomalies were then computed as the ratio (or relative difference) between the monthly Chl-a and the corresponding monthly climatology Chl-a. As an index for the BIOD event, the BDMIs in each month were then calculated^8^.

In addition to satellite-measured Chl-a data, DMI, wind, SST and subsurface temperature, and vertical velocity data were also obtained. The monthly surface wind data were generated from National Centers for Environmental Prediction (NCEP)/National Center for Atmospheric Research (NCAR)^33^. It has a spatial resolution of 2.5°. The NOAA daily high-resolution-blended SST data with a 1/4° resolution were produced with multi-satellite sensors data using the optimum interpolation (OI) technique^34^. The monthly SST data were generated as the average of the daily SST at each location.

The NCEP Global Ocean Data Assimilation System (GODAS)^35,36^ is a real-time ocean analysis and reanalysis system. It produces subsurface temperature, salinity, velocities at 40 levels in the depth range between 5 m and 4478 m for the temperature and salinity, and the depth range between 10 m and 4736 m for the vertical velocity with a 1/3 × 1/3 degree resolution for all latitudes.

In the period from 2012 to 2022, the monthly climatology of wind, SST, subsurface temperature, and vertical velocity at the 40 layers were calculated as the means of these ocean parameters in a certain month. The monthly climatology 20 °C Isothermal (ISO20) depth at each location was also derived from the climatology monthly temperature profile. Using the monthly climatology SST, vertical velocity profiles, and ISO20 depths as references, we calculated the anomalies for SST, vertical velocities, and ISO20 depths in each month to characterize and quantify the negative IOD event, assess the connection between the physical and biological dynamics in the EIO region, and further explore the driving mechanism for the 2022 negative BIOD event.

## Results

### The 2022 negative BIOD event

#### Biological activity in the 2022 autumn

As indicators for a IOD/BIOD event, DMI and BDMIs can be used to identify, characterize, and quantify the occurrence and strength of the IOD event even though DMI reflects SST change of the IOD event, while the BDMIs can not only represent biological activity in the two dipolar zones but also show the subsurface changes in the EIO region^8^.

**Figure 1:**
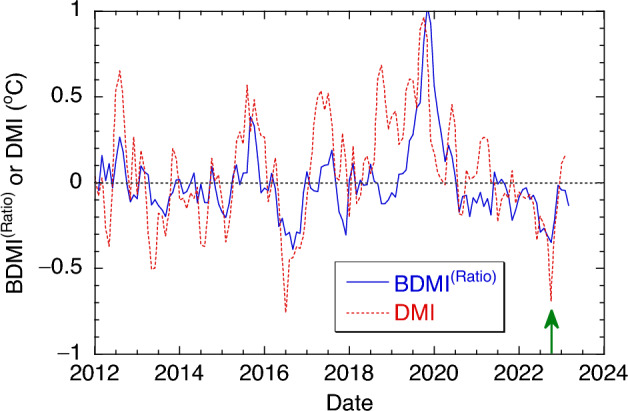
Temporal variations of the DMI and BDMI^(Ratio)^ between January 2012 and March 2023.

Figure 1 shows the variations of DMI and BDMI^(Ratio)^ between 2012 and 2022. It is noted that the seasonal trends in SST and Chl-a are effectively removed when DMI and BDMI^(Ratio)^ are computed in the east and west IOD zones. In general, the BDMI^(Ratio)^ variation is consistent with that of DMI, i.e., high BDMI^(Ratio)^ accompanies high DMI and vice versa. It is noted that DMI and BDMI^(Ratio)^ are defined with the dipolar features of SST and Chl-a in the eastern EIO and western EIO in Saji and Yamagata^16^ and Shi and Wang^8^, respectively.

Specifically, a significant positive BIOD/IOD event occurred in the autumn of 2019. The BDMI^(Ratio)^ reached the maximum of + 0.98 in November 2019, while DMI peaked at + 0.96 °C in October 2019. The positive BIOD event lasted until the spring of 2020. This significantly positive BIOD/IOD event was reported in Shi and Wang^7^. In this period, a negative BIOD/IOD event also occurred in the late summer and early autumn of 2016^8,13^ with BDMI^(Ratio)^ at − 0.39 and DMI at − 0.75 °C. The time lag of the BDMI^(Ratio)^ and DMI represents the different ocean processes in the ocean’s surface and subsurface^8^. The BDMI^(Ratio)^ represents the subsurface biological variation and thermocline dynamics, while the DMI depicts the temperature changes at the sea surface. This leads to the time lag of the DMI in comparison to the BDMI^(Ratio)^ by about one month in the 2019 IOD event.

During the late summer and early autumn of 2022, the EIO experienced another notable negative BIOD/IOD event. The DMI was − 0.25 °C and − 0.32 °C in August and September 2022, respectively. It further dropped to − 0.69 °C in October. This low DMI was comparable to the DMI in the negative IOD event in 2016. In November 2022, the DMI bounced to − 0.27 °C, and back to normal of − 0.092 °C in December. Examination of SST in the EIO region shows the SST anomaly in the east IOD zone (90°E–110°E and 10°S–0°N) were + 0.227 °C, + 0.342 °C, and + 0.134 °C in September, October, and November 2022, respectively, while the corresponding SST anomaly in the west IOD zone (50°E–70°E and 10°S–10°N) were − 0.095 °C, − 0.349 °C, and − 0.135 °C, respectively (https://psl.noaa.gov/gcos_wgsp/Timeseries/DMI/). In fact, the SST changes in the eastern and western EIO clearly showed the negative dipolar SST feature of the EIO in the late summer and autumn of 2022.

In the late summer and autumn of 2022, BDMI^(Ratio)^ also showed that a BIOD event occurred in the EIO. The BDMI^(Ratio)^ was − 0.11 in June, and it began to drop in July and reached − 0.35 in October 2022. The BDMI^(Ratio)^ recovered to − 0.22 in November and − 0.02 in December. In the east IOD zone, the BDMI^(Ratio)^-E were 0.73 and 0.75 in September and October 2022, respectively, while the corresponding BDMI^(Ratio)^-W were 1.04 and 1.10, respectively. This implies that the negative dipolar biological event occurred with enhanced biological activity in the western EIO and depressed biological activity in the eastern EIO in the period of late summer and autumn of 2022.

**Figure 2:**
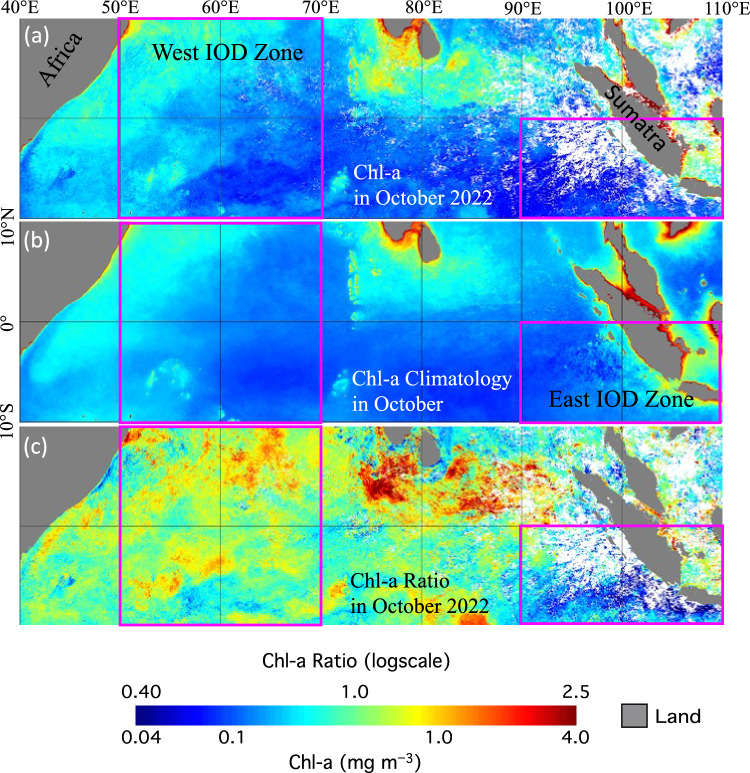
VIIRS-derived ocean color images for (**a**) Chl-a in October 2022, (**b**) Chl-a monthly climatology in October, and (**c**) Chl-a ratio (between panels **a** and **b**) in October 2022. Note that the east IOD zone (90°E‒110°E, 10°S‒0°N) and west IOD zone (50°E‒70°E, 10°S‒10°N) are marked in each panel.

Figure 2 shows the negative BIOD event in October 2022. In October 2022, Chl-a in the east IOD zone (Fig. 2a) were notably lower than the climatology Chl-a in the same month (Fig. 2b) in the offshore region of the west Sumatra Coast even though a large portion of the southeastern east IOD zone was covered with cloud. Chl-a ratio values (between Chl-a in October 2022 and the corresponding October Chl-a climatology) were at 0.4–0.5 in the vast region of the east IOD zone (Fig. 2c).

The contrasting Chl-a change happened in the western EIO. Enhanced Chl-a (Fig. 2a) can be found for most of the west IOD zone during October 2022 in comparison to the Chl-a climatology in the same month (Fig. 2b). In the northern west IOD zone, areas with moderate Chl-a were observed to further expand to the east in October 2022. The Chl-a ratios (between Chl-a in October 2022 and Chl-a climatology in October) were > 1.0 for most of the west IOD zone (Fig. 2c). Note that because Chl-a values were small in October 2022, Chl-a ratios showed clear high patches while these patches were not seen clearly in Chl-a image. In fact, the Chl-a ratio reached ~ 1.4–1.6 in the northern west IOD zone. This shows that Chl-a were indeed enhanced in the west IOD zone in this negative IOD/BIOD event. It is also noted that Chl-a were also enhanced in the central EIO, especially in the northern part of the central EIO during the 2022 negative BIOD event. In contrast, Chl-a were actually depressed in comparison to the climatology Chl-a in the central EIO during the 2019 positive BIOD event^8^.

#### Physical properties in the autumn of 2022

**Figure 3:**
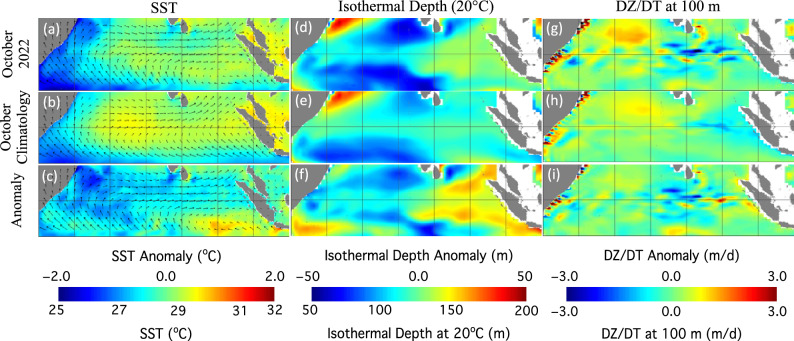
Images of (**a,d,g**) SST, ISO20 depth, and DZ/DT(100) in October 2022, (**b,e,h**) SST climatology, ISO20 depth climatology, and DZ/DT(100) climatology in October, and (**c,f,i**) SST anomaly, ISO20 depth anomaly, and DZ/DT(100) anomaly in October 2022. Note that the wind field in October 2022 is overlaid in (**a,c**), and wind field climatology in October is overlaid in (**b**).

The BIOD event was driven by the variability of physical processes in the EIO. Figure 3 shows various ocean properties, i.e., SST, ISO20 depth, vertical velocity at 100 m depth (DT/DZ(100)), and wind field in October 2022. The comparison of SST in October 2022 (Fig. 3a) and the same-month SST climatology (Fig. 3b) shows that SST in the western EIO in October 2022 were broadly lower than those of the monthly climatology SST. The core of the elevated SST in the west IOD zone in the monthly climatology SST dispersed in October 2022. SST (Fig. 3c) in the west IOD zone, especially in the northern west IOD zone, were ~ 1–2 °C lower than those of the monthly climatology SST. The SST anomaly spatial pattern and coverage in the west IOD zone (Fig. 3c) matched well with the enhanced Chl-a in October 2022 in Fig. 2c.

In the east IOD zone, alongshore winds off the west Sumatra Coast (Fig. 3a) weakened significantly in October 2022 in comparison to the climatology winds in the same month (Fig. 3b). The weak wind could lead to depressed upwelling in the east IOD zone. SST in the east IOD zone in October 2022 showed broad increase in comparison to the corresponding climatology SST. Indeed, in the southern east IOD zone, the SST anomaly was >  + 1.0 °C (Fig. 3c).

As an indicator of the subsurface processes in the EIO, the map of the ISO20 depth (Fig. 3d) in October 2022 was significantly different from the corresponding monthly climatology ISO20 depth (Fig. 3e). In the west IOD zone, ISO20 depth shoaled up ~ 10–40 m in the northern west IOD zone (Fig. 3f), while the ISO20 depth deepened ~ 10–30 m in the eastern EIO region (Fig. 3f). In most part of the east IOD zone, the ISO20 depth deepened by ~ 20–30 m.

The enhanced positive DZ/DT(100) anomaly in the northern west IOD zone was observed in October 2022 (Fig. 3g). This was generally consistent with the cooler SST (Fig. 3a,c) and shoaling ISO20 depth (Fig. 3d,f). In the eastern Equatorial Pacific Ocean, the DZ/DT(100) generally were weaker in October 2022 (Fig. 3g) than those of the corresponding climatology DZ/DT(100) (Fig. 3h). The discrepancy of the spatial pattern of the DZ/DT(100) anomaly (Fig. 3i), the SST anomaly (Fig. 3c), and the ISO20 depth anomaly (Fig. 3f) in the eastern EIO could be attributed to the complication of the ocean processes such as wind-driven upwelling, ocean current, and propagation of the planetary wave, etc.

### Connection between the physical and biological variability

The IOD/BIOD events in the EIO are featured with contrasting physical and biological variabilities in the east and west IOD zones. To further characterize the driving mechanism for both the positive and negative IOD/BIOD events and their connections with the physical processes, we further analyzed the anomalies of Chl-a, SST, ISO20 depth, and DZ/DT(100) in the period between January 2018 and March 2023 in the east and west IOD zones, respectively. In this period, the EIO region experienced a significant positive IOD/BIOD event in the autumn 2019 and a moderate negative IOD/BIOD event in 2022 as shown in Fig. 1.

#### The east IOD zone

**Figure 4:**
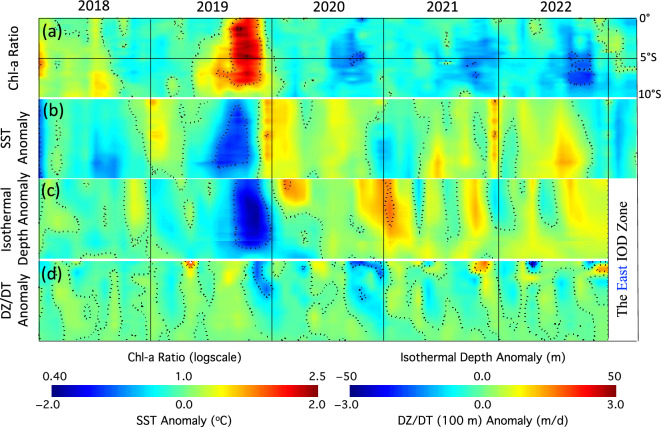
Time series of longitudinal average values in the east IOD zone between January 2018 and March 2023 for (**a**) Chl-a ratio, (**b**) SST anomaly, (**c**) ISO20 depth anomaly, and (**d**) DZ/DT(100) anomaly.

In the east IOD zone, the longitudinal averages of Chl-a ratio, SST anomaly, ISO20 depth anomaly, and DZ/DT(100) anomaly were computed (Fig. 4). The Chl-a ratio (Fig. 4a) clearly showed significantly enhanced values in the 2019 autumn. In fact, the Chl-a ratio reached >  ~ 2.5 between the equator and 5°S. Most of the anomalous Chl-a were located in 0°–8°S. In the autumns of 2020 and 2021, Chl-a ratio values were slightly < 1.0, consistent with those of the BDMI^(Ratio)^ (Fig. 1) and BDMI^(Ratio)^-E (not shown here). In the autumn of 2022, moderate decrease of the Chl-a ratio was observed in the region south of 5°S. The coverage and the location of the anomalous low Chl-a in the autumn of 2022 were apparently different from those of the anomalous high Chl-a in the 2019 positive BIOD event in the east IOD zone. Figure 4a indeed demonstrates the contrasting difference of Chl-a in the east IOD zone during the positive BIOD event in 2019 and negative BIOD event in 2022.

The anomalies of SST (Fig. 4b), ISO20 depth (Fig. 4c), and DZ/DT(100) (Fig. 4d) generally showed consistence with those of Chl-a ratio for both the positive and negative IOD/BIOD events from 2018 to 2023. In the autumn of 2019, SST dropped by ~ 1.5 °C from equator to 8°S, while SST increased moderately by ~ 1 °C in the southern east IOD zone in late summer and early autumn of 2022 (Fig. 4b). The ISO20 depth shoaled up by ~ 50 m in the autumn of 2019 and it deepened by ~ 20–30 m in the 2022 negative IOD/BIOD event (Fig. 4c). The connection of DZ/DT(100) anomaly and Chl-a ratio was less significant than that between Chl-a ratio and SST anomaly or ISO20 depth anomaly even though positive DZ/DT(100) anomaly indeed occurred in the positive 2019 IOD event, while there was negative DZ/DT(100) anomaly in the negative 2022 IOD event.

It is noted that the ISO20 depth anomaly for the positive BIOD in 2019 and negative BIOD in 2022 coincided and collocated with the Chl-a ratio in the same period, while the Chl-a ratio lagged the SST anomaly for about one month. This indeed suggests that BDMI^(Ratio)^, which was calculated as the difference between Chl-a ratios in the east and west IOD zones, reflected the subsurface variability. In the 2019 positive IOD event, strengthened southeasterly winds alongshore off the west Sumatra Coast^7^ in the east IOD zone caused strong offshore upwelling and lifted the ISO20 depth, while the decreased coastal upwelling and deepening ISO20 depth were attributed to the weakened alongshore wind during the negative BIOD event in the autumn of 2022.

#### The west IOD zone

**Figure 5:**
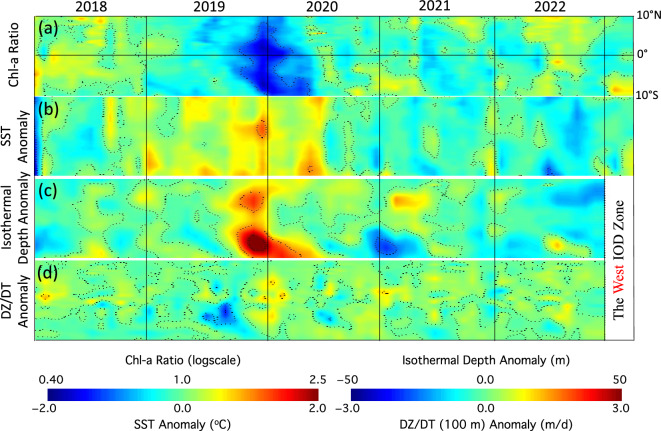
Time series of longitudinal average values in the west IOD zone between January 2018 and March 2023 for (**a**) Chl-a ratio, (**b**) SST anomaly, (**c**) ISO20 depth anomaly, and (**d**) DZ/DT(100) anomaly.

Figure 5 shows the longitudinal averages of Chl-a ratio (Fig. 5a), SST anomaly (Fig. 5b), ISO20 depth anomaly (Fig. 5c), and DZ/DT(100) anomaly (Fig. 5d) in the west IOD zone between January 2018 and March 2023. Indeed, both the Chl-a ratio (Fig. 5a) and SST anomaly (Fig. 5b) showed the dipolar features in comparison to the Chl-a ratio (Fig. 4a) and SST anomaly (Fig. 4b) during the 2019 positive IOD/BIOD event and 2022 negative IOD/BIOD event. During the late autumn of 2019, the Chl-a ratio dropped to ~ 0.6. Low Chl-a were mainly in the southern west IOD zone, and the anomalously low Chl-a lasted from the autumn 2019 to early spring of 2020 (Fig. 5a). In contrast, the anomalous high Chl-a occurred in the autumn of 2022 with Chl-a ratio at ~ 1.2–1.3. Most area of the enhanced Chl-a were in the northern east IOD zone from 0° to 10°N.

The SST and ISO20 depth anomalies in this period (Fig. 5b,c) showed the feature of the physical IOD, i.e., higher SST with deepening ISO20 depth during the positive IOD event in 2019 and lower SST with shoaling ISO20 depth during the negative IOD event in 2022. Specifically, the ISO20 depth deepened over ~ 50 m in the November and December 2019 at 7°–9°S of the west IOD zone. It is also noted that the Chl-a ratio and ISO20 depth anomaly generally coincided and collocated in the west IOD zone for both the positive (2019) and negative (2022) IOD/BIOD events. In the autumn of 2019, the enhanced deepening of the ISO20 depth between late 2019 and early 2020 in the southern west IOD zone also came with even lower Chl-a ratio in that region. On the other hand, the shoaling ISO20 depth between August and December 2022 in the northern west IOD zone also matched well with the higher Chl-a in that region during the same period.

The DZ/DT(100) anomaly (Fig. 5d) generally matched the Chl-a anomaly in the west IOD zone in the 2019 positive and 2022 negative IOD/BIOD events even though the patterns of Chl-a ratio (Fig. 5a) and DZ/DT(100) anomaly (Fig. 5d) were not similar. Specifically, the DZ/DT(100) anomaly of ~  − 1 to 2 m/day was observed over the region of 0°–5°S in the west IOD zone during the early autumn of 2019. In the autumn of 2022, the DZ/DT(100) anomaly was ~  + 0.5 m/day from August until December 2022 in the northern west IOD zone.

### Physical and biological processes of the positive and negative BIOD events

Nutrient dynamics, e.g., nitrate, phosphate, is critical in the ocean biological variability and plays as a connection between a physical IOD and its corresponding BIOD event. In the EIO region, the World Ocean Atlas 2018 (WOA18) field data showed that nutrient concentrations became high with the increase of water depth (https://www.ncei.noaa.gov/access/world-Ocean-atlas-2018/), and sharp increase could be found in the thermocline depth with the nitrate concentration about 10–20-fold of that at the sea surface^37^.

Shi and Wang^7^ showed that stronger upwelling-favorable southeasterly winds along the west Sumatra Coast led to the enhanced upwelling and shoaling of the thermocline, brought up nutrient rich subsurface water to the surface and consequently triggered phytoplankton bloom in the east IOD zone in the autumn of 2019. In the west IOD zone, the lower Chl-a during the autumn of 2019 was attributed to the dowelling, Ekman pumping, and surface water convergence that led to deepening thermocline depth, higher SST, depleted sea surface nutrient level, and depressed Chl-a in the west IOD zone. Figures 4 and 5 provide further evidence that the ISO20 depth and Chl-a anomalies coincided and collocated during the positive IOD/BIOD event in 2019 for both the east and west IOD zones. Indeed, the positive BIOD in 2019 was driven by the sea surface nutrient variability caused by the subsurface physical dynamics.

This study provides us an opportunity to get an insight of the biological and physical variability during a negative IOD/BIOD event. The Chl-a anomaly in both the east and west IOD zones generally coincided and collocated with the ISO20 depth and SST anomalies, i.e., depressed Chl-a with deepening thermocline in the east IOD zone and increased Chl-a with shoaling thermocline in the west IOD zone. The algal growth and phytoplankton bloom are driven by nutrient dynamics such as nitrate and phosphate. In the EIO, nutrient concentrations increase significantly with the increase of water depth^37^. As an example, nitrate concentration increases from ~ 0.5 μmol/kg at the surface to > 10 μmol/kg at the bottom of the thermocline in the central east IOD zone. This also implies that the lower sea surface nutrient levels driven by the weaker upwelling and deepening thermocline in the east IOD zone were the driver for the anomalously low Chl-a in the east IOD zone during the 2022 negative BIOD event. On the other hand, higher sea surface nutrient levels due to the surface water divergence and shoaling thermocline in the west IOD zone led to higher Chl-a in the west IOD zone during the negative BIOD event.

Overall, Figs. 4 and 5 characterize and quantify the IOD-driven Chl-a variability and its connection with SST, ISO20 depth, and subsurface vertical velocity for the positive and negative BIOD events in 2019 and 2022, respectively. In comparison to the charts for BDMI^(Ratio)^ and DMI in Fig. 1, Figs. 4 and 5 show not only the time-series variations of Chl-a anomaly and the corresponding physical variability, but also the spatial distributions of anomalies for Chl-a, SST, ISO20 depth, and DZ/DT(100). These results give us more details such as how the positive and negative BIOD events started, developed, and evolved, as well as where the most significant anomalies occurred. As an example, the depressed Chl-a occurred in almost the entire west IOD zone during the 2019 positive BIOD event, while most of enhanced Chl-a anomaly was in the northern west IOD zone in 2022 negative BIOD event (Fig. 5a). Indeed, these results provide us a comprehensive understanding of how the biological changes during both positive and negative BIOD events were driven by the ocean physical dynamics in the EIO region.

## Discussions and conclusion

This work provides a complementary example and new knowledge about the negative BIOD event and its driving forces. We have studied and quantified extensively the driving forces such as the isothermal depth anomaly, SST anomaly, and anomaly of upwelling speed at 100 m. Different from the report of the BIOD in 2019^7^, the detailed characterizations of how and where the enhanced and suppressed biological activities developed and evolved, as well as the relationships between the biological variability and the physical variability in the two IOD zones, are provided in this work. These results are important and useful to better understand both the positive and negative BIOD events and their driving mechanisms.

In this study, we report an important negative BIOD event in the autumn of 2022. The BIOD index BDMI^(Ratio)^ reached below − 0.31, while IOD index DMI was − 0.69 °C in October 2022. The BDMI can effectively identify this negative BIOD event and characterize the development and strength of this negative BIOD event. The VIIRS images showed that in the autumn of 2022 the depressed and enhanced Chl-a were observed in the east and west IOD zones, respectively. In fact, Chl-a ratio values were ~ 0.4–0.5 in the offshore region along the west Sumatra Coast in the southern east IOD zone, compared with those of ~ 1.4–1.6 in the northern west IOD zone.

In the autumn of 2022, weakened southeasterly winds along the west Sumatra Coast were observed. The time series of longitudinal averages of the Chl-a ratio, SST anomaly, ISO20 depth anomaly, and DZ/DT(100) anomaly between January 2018 and March 2023 provide the details of the progress of the Chl-a anomaly and its connection with the changes of these ocean physical properties. For both the positive (2019) and negative (2022) BIOD events, the Chl-a ratio generally coincided and collocated with the ISO20 depth anomaly. The nutrient dynamics driven by the physical IOD was attributed to both the positive and negative BIOD events. The physical and biological processes and mechanism of the negative BIOD in 2022 were essentially opposite to that for the positive BIOD event in 2019^7^. In the negative IOD event, weakened upwelling led to deepening thermocline depth, decreased sea surface nutrient level, and consequently hampered algae growth in the east IOD zone. In the west IOD zone, the surface water divergence and alleviated Ekman pumping caused shoaling thermocline, elevated sea surface nutrient level, and eventually boosted the phytoplankton growth.

## Data Availability

The datasets used and/or analyzed during the current study are available from the corresponding author on reasonable request.
